# A qualitative study of diphenhydramine injection in Kyrgyz prisons and implications for harm reduction

**DOI:** 10.1186/s12954-020-00435-7

**Published:** 2020-10-31

**Authors:** Jaimie P. Meyer, Gabriel J. Culbert, Lyuba Azbel, Chethan Bachireddy, Ainura Kurmanalieva, Tim Rhodes, Frederick L. Altice

**Affiliations:** 1 Yale School of Medicine, AIDS Program, 135 College Street, Suite 323, New Haven, CT 06510 USA; 2 Population Health Nursing Science, College of Nursing, University of Illinois at Chicago, Chicago, IL USA; 3 Virginia Commonwealth University School of Medicine, Richmond, VA USA; 4 AIDS Foundation East-West, Bishkek, Kyrgyzstan; 5 The London School of Tropical Hygiene and Tropical Medicine, London, UK

**Keywords:** Antihistamines, Eastern Europe and Central Asia (EECA), HIV, Injection drug use, Methadone, Prisons

## Abstract

**Background:**

To reduce opioid dependence and HIV transmission, Kyrgyzstan has introduced methadone maintenance therapy and needle/syringe programs into prisons. Illicit injection of diphenhydramine, an antihistamine branded as Dimedrol^®^, has been anecdotally reported as a potential challenge to harm reduction efforts in prisons but has not been studied systematically.

**Methods:**

We conducted qualitative interviews in Kyrgyz or Russian with prisoners (*n* = 49), former prisoners (*n* = 19), and stakeholders (*n* = 18), including prison administrators and prisoner advocates near Bishkek, Kyrgyzstan from October 2016 to September 2018. Interviews explored social–contextual factors influencing methadone utilization in prisons. Transcripts were coded by five researchers using content analysis. Dimedrol injection emerged as an important topic, prompting a dedicated analysis.

**Results:**

After drinking methadone, some people in prison inject crushed Dimedrol tablets, a non-prescription antihistamine that is banned but obtainable in prison, to achieve a state of euphoria. From the perspectives of the study participants, Dimedrol injection was associated with devastating physical and mental health consequences, including psychosis and skin infections. Moreover, the visible wounds of Dimedrol injecting contributed to the perception of methadone as a harmful drug and supporting preference for heroin over methadone.

**Conclusion:**

Dimedrol injecting is a potentially serious threat to harm reduction and HIV prevention efforts in Kyrgyzstan and elsewhere in the Eastern European and Central Asian region and requires further investigation.

## Background

[He] also died last year. I got his passport done for him. He has a mother and two brothers who sent money through me. I asked him, “What keeps you here?” I said to him, “Come on, I’ll take you to detox and you’ll spend ten days there and you’ll have a ticket, and I’ll take you straight to [a rehabilitation center]…What keeps you here is Dimedrol only, nothing else. You’ve exchanged your relatives for Dimedrol!” He didn’t go and he ended up dying in a manhole. He shot up in the groin. Everything rotted there. And we called the police officers. They took him out of the manhole already semi-decomposed. [Marina]

HIV incidence is declining globally, with many countries poised to achieve UNAIDS goals of zero new HIV infections by 2030 [1]. Yet, HIV incidence is increasing in Eastern Europe and Central Asia (EECA) where drug injection is the primary driver of HIV transmission within the community. In the EECA region, HIV prevalence among people who inject drugs (PWID) is 25–30%, compared to 0.8% HIV prevalence in the general adult population [2]. Criminalization of drug use in EECA countries has concentrated people with HIV and substance use disorders (SUDs) into jails and prisons. Kyrgyzstan (Kyrgyz Republic) is an EECA country of six million people with high HIV prevalence (11.3%) among prisoners and detainees [3], most of whom (95%) are male [4] and one-third of whom are PWID [5, 6]. From 2000 to 2018, Kyrgyzstan’s prison population declined by half, from 20,000 to approximately 10,000 detainees; however, seizures of illicit drugs and substance use within prisons increased during that same period. In 2012, two percent of all drug-related crime nationwide occurred in prisons [7], evidencing the need for a coordinated and comprehensive response to prevent, treat, and mitigate the harmful consequences of substance use within Kyrgyz prisons.

To address ongoing substance use and high HIV prevalence within its prisons, Kyrgyzstan implemented a comprehensive package of harm reduction and HIV prevention services in prisons as recommended by the United Nations Offices on Drugs and Crime (UNODC) and AIDS (UNAIDS), and the World Health Organization (WHO) [8]. In 2001, Kyrgyzstan introduced needle syringe programs (NSP) into prisons, followed by a pilot methadone maintenance program (MMT) in 2008. Despite availability of MMT and NSP in most of Kyrgyzstan’s 28 prisons, which together provide pharmacological treatment with methadone to more than 400 individual patients [9], utilization of MMT remains suboptimal and many people continue to use and inject drugs within prison [6]. NSP, which could reduce harmful consequences of drug injection, is not routinely offered to prisoners receiving MMT because they are assumed to be abstinent from heroin. Major barriers to MMT and NSP include stigma among prisoners and staff [10], restricted access, and a competing heroin market controlled by the informal prison leadership, as we have described elsewhere [6].

Illicit injection of diphenhydramine in prison has been noted in government reports since 2006, but has not been assessed as a specific threat to individual patient health or MMT service expansion [11, 12]. Diphenhydramine is an H1 antihistamine branded as Dimedrol^®^ in Kyrgyzstan. Though it has an opioid-sparing effect, Dimedrol readily crosses the blood–brain barrier into the central nervous system, resulting in predominantly sedative and antiemetic effects [13]. As a class, antihistamines may theoretically have a synergistic effect with opioids because they inhibit opioid metabolism [14]. Antihistamines’ affinity for the dopamine receptor and potential to increase dopamine release, at least in vitro, may lead PWID to use it to “potentiate euphoria” with opioids or opioid agonists [15]. The combination of opioids with antihistamines has been a recurrent global trend, often noted in settings where MMT or heroin availability is scarce. For example, combined opioids and antihistamines were commonly known as a “lytic cocktail” in 1950s France, “Blue Velvet” in the US in the 1960s, and “T’s and Blues” in the US in the 1980s [16]. Illicit antihistamine use has more recently been reported among patients receiving MMT in San Francisco [17] and among US patients receiving methadone for chronic pain [18]. The gray literature includes reports of Dimedrol injecting throughout the EECA among people in the community with opioid use disorders, including in Tajikistan [19], Russia [20], and Uzbekistan [21].

Dimedrol injecting is potentially detrimental to harm reduction and HIV prevention efforts, particularly in Kyrgyz prisons where HIV prevalence is high and methadone remains unpopular despite its availability for over a decade. We have previously described how the low uptake of methadone in Kyrgyz prisons is related to how other drugs are used in this context [22]. Despite its persistent global popularity, antihistamine injecting has never been specifically addressed in terms of HIV prevention, harm reduction, or public health. In this qualitative analysis, we describe Dimedrol injecting practices in Kyrgyz prisons and explore its health consequences, including as a possible influence upon prisoners’ acceptance and utilization of MMT.

## Methods

### Study setting

This study was conducted near Bishkek, Kyrgyzstan from October 2016 to September 2018. In Kyrgyz prisons, MMT is provided by the prison administration and delivered by trained medical staff to persons meeting DSM-IV criteria for opioid use disorder (OUD). In recognition of the availability of heroin in prison, NSP is also offered by the prison administration and available to any prisoner who requests sterile equipment for injecting. Within Kyrgyz prisons, an informal caste system is organized around prison labor and narcotic distribution. Those at the highest level of the prison hierarchy are informal prison leaders who dictate codes of conduct. Caste is assigned by compliance with these codes of conduct and organized around contribution of labor to the *[obshchak]*, or prisoners’ common fund. Participation in the prisoner economy is rewarded with distribution of *[razgon],* or material rewards including heroin, which is trafficked and controlled by informal prison leadership as described elsewhere [23].

The parent project was designed to longitudinally and qualitatively assess barriers to MMT uptake during and after incarceration and evaluate the ways in which the prison risk environment influenced perceptions and use of methadone. Findings on HIV risk [24] suggest that most PWID incarcerated in Kyrgyzstan continue to inject in prison [6], prisoners are organized into rigid hierarchies that perpetuate inequalities in access to HIV prevention resources [23], and social factors shape patient engagement with methadone [22], especially after prison release and during the transition to communities [25] and among women [26]. Here, we explore the harmful health effects of Dimedrol injecting in prisons and implications for harm reduction programs.

### Participant recruitment

**Fig. 1:**
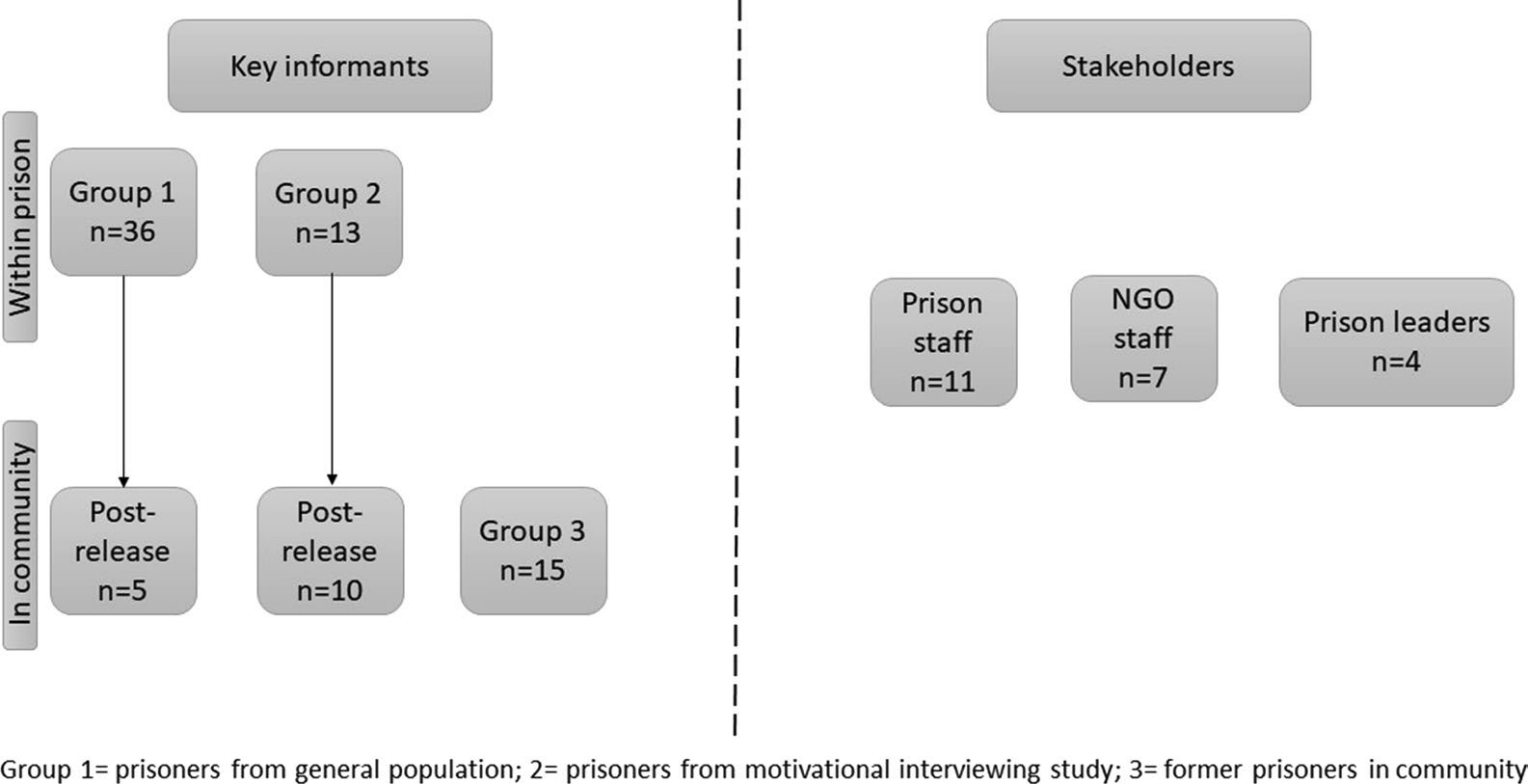
Study sampling overview

Participants were recruited from Kyrgyzstan’s largest male prison, which houses more than 1000 men, and Kyrgyzstan’s only female prison, which houses approximately 280 women. Studies with nationally representative cohorts indicate higher prevalence of HIV among male prisoners (11.3%) compared to female prisoners (2.5%) [5], and male prisoners reporting higher rates of substance use within prison [6]. Eligible participants were 18 years of age or older, within 6–12 months of scheduled prison release, reported having injected opioids in the 12-months prior to incarceration, and planned to return to the Bishkek region on release. We used purposive sampling to recruit people from the soon-to-be released prison population who were diverse with respect to age, incarceration history, HIV status, and prior experiences with methadone [5] (Fig. 1: Group 1). We also recruited participants from a concurrent NIDA-funded implementation study of motivational interviewing for MMT initiation and retention through the post-release transitional period (Fig. 1: Group 2). Group 2 participants were only recruited for the qualitative study after they had completed the baseline motivational interview visit and declared their preferences for methadone; the same eligibility criteria were applied as to Group 1. To elicit accounts of within-prison substance use in an environment free from the constraints of the prison (i.e., time restrictions, pressures of informal prisoner subculture, etc.), we recruited additional participants from community organizations in Bishkek who reported lifetime history of incarceration in Kyrgyzstan (Fig. 1: Group 3). Additionally, we recruited stakeholders for interviews, including: (1) prison staff employed by the State Penitentiary Service; (2) staff from non-governmental organizations who serve as advocates for returning prisoners; and (3) formerly incarcerated individuals who were identified as leaders within prisoner subculture (Fig. 1).

### Study procedures

All participants completed informed consent procedures and a face-to-face, voice-recorded in-depth interview in Kyrgyz or Russian with a trained interviewer. Interviews were conducted in private rooms within each of the prison facilities and lasted ~ 30–60 min. Prison staff were never present during interviews. Interviews focused on social, interpersonal, and environmental factors that influence drug-related HIV-risk behaviors and participation in methadone treatment within prison and during community reentry. A topic guide covered the following central issues: social support, within-prison drug use, knowledge about and attitudes toward drug treatment, and interactions with other prisoners and prison staff. Interviews with prison staff sought to understand the prisoner social hierarchy (caste system) and economy, MMT, NSP, within-prison drug use, and perceptions of “us” (e.g., prisoners) versus “them” (e.g., prison staff or administration). Interviews were open-ended, and the topic guide was modified to incorporate emerging themes of interest, including Dimedrol. Because this study was designed as a longitudinal qualitative assessment, we conducted one interview per participant in prison prior to release and aimed to conduct one follow-up interview per participant in the community following release, using a similar (post-release) interview guide, which focused additionally on substance use in the community (Fig. 1).

### Analysis

Voice-recorded interviews were transcribed verbatim and translated into English using a HIPAA-compliant service. Interview transcripts were also analyzed in Russian because several study team members (LA, JR, AK) are native Russian speakers, allowing for interpretation of the participant data in its original form. All identifying information was removed before transcripts were transferred for analysis. All transcripts were uploaded into Dedoose qualitative analytic software. Using an inductive approach, five researchers independently coded the interviews using a shared codebook. Initial codes were informed by concepts of the risk environment framework, including *form of environmental influence* and *level of effect* [27]. The codebook was then adapted as part of an iterative process to allow for “open coding” of participant narratives. For the present analysis, all excerpts from the parent code “Dimedrol” were examined, including: *meanings of, descriptions of people who use, addiction, intoxication from, attitudes toward people who use, prevalence of use, procedures of using, reasons for using, side effects from, relationship to methadone, polysubstance use with methadone, and polysubstance use with heroin.* Excerpts were grouped around key themes based on study team consensus and discussion and illustrative quotes were selected for each. Participants are identified by pseudonyms as per sociology discipline convention and consistent with pseudonyms used in prior analyses from the parent study [28].

## Results

Participant characteristics are shown in Table 1. Although qualitative data were drawn from interviews with nearly 20 women in prison, representative excerpts from only one woman are included here because Dimedrol use was less common in the women’s prison where few women prisoners are prescribed methadone. People using MMT in prison were said to frequently combine methadone with Dimedrol to achieve a euphoria that was not possible with methadone alone. The major motivation for Dimedrol use was the belief that it increased the activity of methadone and strengthened its effects:

Dimedrol gives a push, they inject it to increase the effect of methadone, to get high. [Bashir]

I think it is stronger, more potent that heroin. Methadone and Dimedrol, in my opinion, are more potent. [Yryskul’]

Some participants suggested that Dimedrol use originated in the community where people injected low doses of Dimedrol to reduce nausea after heroin injection or injected Dimedrol in higher doses to strengthen the effects of low purity heroin:

It depends on the body of each and the heroin. Generally, you don’t find good heroin outside. In my days, a gram of heroin cost 1000 rubles. You take this gram, but only one tenth of it is heroin and nine tenths are sugar. We were shooting up sugar, and not heroin, and by adding [Dimedrol] tablets, it added strength. [Tursun]

Whereas Dimedrol was sometimes combined with heroin in the community, in prison, Dimedrol was only ever identified as being combined with methadone.

**Table 1:** Table of attributes

Pseudonym	Role/status in hierarchy	Gender	Age range	Duration PWID	Years on MMT	Lifetime # incarcerations	Duration current incarceration (y)
*Key informants*							
Bashir	Obizhennyi^a^	M	26–30	1–5	0	3	5–6
Yryskul’	NA	F	31–35	6–10	0	2	1–2
Tursun	Muzhik/poriadochnyi^b^	M	46–50	1–5	0	1	3–4
Turat	Muzhik/poriadochnyi	M	41–45	16–20	5	6	1–2
Sasha	Muzhik/poriadochnyi	M	56–60	26–30	> 10	> 7	3–4
Nikolay	Reds^c^	M	46–50	21–25	3	4	1–2
Tair	Muzhik/poriadochnyi	M	46–50	36–40	0	7	5–6
Envar	Muzhik/poriadochnyi	M	31–35	16–20	0	4	1–2
Iurii	Muzhik/poriadochnyi	M	31–35	31–35	> 1	4	> 8
Daniyar	Gady^d^	M	41–45	21–25	1	4	3–4
Yuri	Muzhik/poriadochnyi	M	36–40	6–10	0	7	3–4
*Key stakeholders*							
Marina	NGO staff	F					
Timur	Blatnoi^e^	M					
Oksana	Women’s prison staff	F					
Nurgul’	Medical staff	F					

*PWID *person who injects drugs, *MMT *methadone maintenance therapy, *NA *not applicable, *M *male, *F *female, *NGO* non-governmental organization

^a^The lowest caste of prisoners

^b^Literally, “the decent ones.” Second to highest and largest caste in the prisoner hierarchy

^c^Refers to prisoners who work for the formal prison administration

^d^The second to lowest caste in the prisoner hierarchy

^e^One of the higher castes in the prisoner hierarchy, a leader within prison subculture

### Health consequences of Dimedrol use: “They are walking around like zombies”

Participants frequently and vividly described Dimedrol injection as resulting in a range of negative mental and physical health effects. People who combined methadone and Dimedrol were described as becoming progressively withdrawn, forgetful, and confused:

Well, they would not answer your questions, sometimes they would talk nonsense and gibberish. Their eyes are crazy. I believe I heard someone say they would start going through garbage, or even put their hands in the toilet bowls. In a nutshell, people become just awful. [Turat]

I have no idea what they put in Dimedrol… but it makes you stall, unresponsive, it is hard to explain. It has stronger highs than heroin. [Yryskul’]

In addition to these altered behaviors, Dimedrol use was associated with skin and soft tissue infections, including abscesses and chronic wounds:

For so many years I had veins, when I started shooting up Dimedrol, in a matter of six months, all my veins were gone, in the groin and in my armpits. All the things this Dimedrol has done, I almost lost my arm. [Sasha]

Participants described Dimedrol as short-acting and leading to tolerance, which required users to inject it in larger quantities and more frequently:

Before methadone, you shoot up five tablets. You drink methadone some 20 min later, it’s absorbed and you crush ten tablets of Dimedrol and you shoot up ten tablets, and it’s euphoria. An hour later another ten tablets. You do 50 tablets a day, can you imagine? [Sasha]

In addition to causing pain and disability, Dimedrol injection was described as visibly altering a person’s physical appearance and behaviors in ways that marked them as members of a hopelessly self-destructive caste scorned by medical staff and other prisoners:

Well those who take Dimedrol, it’s obvious. They shoot up, then they get abscesses. They’re always walking around in bandages. You can pick out such a person straight away. [Nikolay]

All of them are rotting, and such a smell, they really care little about this, how they smell and how they look. [Marina]

A guy here has already had his finger cut off. Now the second time his leg was cut off. The third time, I said, let them cut his head off and be done with it. [Tair]

Their behavior becomes different, yes. When they’re taking methadone, the person behaves exactly like you and me. But when it’s drugs [Dimedrol], if she’s taking something on top [of the methadone], this means that her behavior changes radically, as it is with drug addicts. [Oksana]

Although people who inject Dimedrol in prisons theoretically have access to the NSP, there are severe social sanctions against disclosing Dimedrol use, described further below. As a result, most people who inject heroin in prison do so with sterile injecting equipment, whereas people who inject Dimedrol may have limited access to NSP and frequently share injecting equipment, with potential risk for transmission of blood-borne infections including HIV. Participants were divided about whether prisoners receiving MMT could access sterile syringes for injecting Dimedrol. Some participants felt that prisoners who used Dimedrol could access sterile syringes under the pretext of using needles to self-administer other medications.

They do not say that they take it for Dimedrol, they say something else. It is prohibited. But they will give syringes for medicines. [Bashir]

Other participants felt that prisoners who used Dimedrol could obtain sterile syringes from other prisoners.

If they’re on methadone, they [sterile syringes] are not given, but maybe they’re getting them through some friends. [Daniyar]

### Social consequences of Dimedrol use within prison

Prisoner leaders, by dictating codes of conduct and determining participation in the *obshchak*, played a main role in regulating the use of Dimedrol. When MMT was first introduced in the prison, prisoner leaders noted a sharp increase in Dimedrol-associated deaths and delivered a decree *[progon]* that banned Dimedrol use, especially by members of the middle prisoner caste *[poriadochnye]*.

When Dimedrol was sold, there were many cases of deaths. After that, they prohibited trading, the sale of Dimedrol. They had a corpse there each week. [Nikolay]

It’s banned, bones rot from Dimedrol, and there are talks that if anyone finds out about Dimedrol, bad things would happen, Dimedrol is banned. [Tursun]

One major consequence of the Dimedrol ban is that people who inject Dimedrol must do so furtively. Fears of repercussions cause people to delay seeking care from prison health services for injection-related skin and soft tissue infections:

He shoots Dimedrol. He misses the vein, an abscess forms. You know what that is. He rots. He doesn’t treat it promptly because there’s a special punishment for doing Dimedrol. [Tair]

Although some participants perceived that the prohibition on Dimedrol was motivated by the informal leaders’ sense of responsibility for the welfare of prisoners, others thought the true motivation was to preserve a functional and compliant labor pool:

When there is Dimedrol involved, one cannot function properly, one cannot work, one will just be high. [Turat]

Stigmatization of people who use Dimedrol by other prisoners and members of the informal prison leadership compounded stigmatization of people who accessed MMT. Because MMT was run by the formal prison authority (as opposed to the heroin trade which is run by the informal prison leadership), prisoners receiving MMT were viewed by other prisoners as being aligned with the formal prison authority and viewed suspiciously by the informal prison leaders and other prisoners. Prisoners utilizing methadone are barred from participating in the *razgon* distribution of heroin by the *obshchak*, which is a cornerstone of the prison leaders’ control.

Dimedrol is also banned by formal prison administrators, who have their own punitive approach to people in prison using Dimedrol:

If the guards catch a patient with Dimedrol, a report is immediately drafted…and the patient is locked up in isolation. [Nurgul’]

### The Dimedrol narrative against methadone

Explanations for the Dimedrol economy drew on prisoners’ shared understandings about the nature of opioid dependence, perceptions of PWID, understandings of methadone, and perceptions of prison authority. People on MMT were perceived as being especially vulnerable because they can lose their status in the prison hierarchy. Despite formal bans on Dimedrol by the prison administration and by the prisoner leaders, people can obtain Dimedrol illicitly: “They get it from the outside.”

Criminal penalties for Dimedrol use provided another layer of social control.

Yes, because leaking information, he’s now, his psychology is under the influence, first, of methadone, second, the cops [formal prison administration], and on the part of the [informal leaders]. Because, if he is discovered by the [informal leaders], he’ll really get it, and if he isn’t discovered, sooner or later everything comes to the surface. [Timur]

In a prison setting where PWID are vulnerable to exploitation and informal and formal leadership systems compete for control, both MMT and Dimedrol acquire meaning that influence their use and distribution [22]. These explanations borrowed heavily from a shared sentiment that methadone was harmful and not to be trusted. As one interviewer recorded in his fieldnotes:

There’s even a rumor going around that Americans thought of Dimedrol to combine it with methadone in order to kill the drug using population. That’s how much they go hand in hand and are seen as something foreign.

Dimedrol is not used on its own and methadone and Dimedrol are often conflated because of how they are used [22]. The harms of Dimedrol use were attributed to methadone and used as an argument for phasing out methadone:

It actually has a lot to do with methadone because, without methadone, for example, why the hell would anyone need this shit? [Envar]

It would be better if they were giving heroin that way [as they distribute methadone]. And they would shoot up less Dimedrol. They’re only sending themselves to their deaths…If there wasn’t methadone, [they] wouldn’t be looking for Dimedrol. [Iurii]

This explicit linking of Dimedrol injection with methadone treatment was evident also in interviews with prison administrators and other non-prisoner participants who acknowledged the difficulty of intervening on Dimedrol injection within Kyrgyz prisons that take a mainly punitive approach to Dimedrol possession.

## Discussion

In this study, we found that Dimedrol injection is a potentially serious drug-related harm among people receiving methadone treatment in Kyrgyz prisons. Participants’ accounts point to how methadone is used alongside other substances (i.e., Dimedrol). The relationship between Dimedrol and methadone contributes to methadone’s unpopularity and low uptake among prisoners within a criminal subculture that benefits from heroin distribution. This study has implications for the further quantitative investigation of antihistamine polysubstance use as well as sociological inquiry into how the relations between different drugs affect the way drugs are used.

In the context of a regional rise in HIV incidence primarily associated with injection drug use, our findings suggest that Dimedrol injecting can no longer be ignored as a public health problem. Dimedrol injection and its health and social consequences were repeatedly raised as areas of concern for MMT implementation by prisoner and non-prisoner participants. In this study, prisoner and non-prisoner participants, including prison administrators and medical staff members, described illicit injection of crushed Dimedrol tablets as a serious and potentially widespread behavioral health concern affecting people receiving methadone treatment in prison. Participant accounts contain graphic descriptions of disfiguring injuries and behavioral changes consistent with possible effects of diphenhydramine injection described elsewhere [17–21]. Participants pointed to these physical and behavioral changes as evidence that methadone was harmful. Heroin, on the other hand, remained untainted by Dimedrol and therefore is a more favorable substance among prisoners adhering to the rules of criminal subculture. Prison administrators, medical staff, and community members recognize and have requested support to address the issue but acknowledge long-standing difficulties in controlling the supply of drugs within prison that have contributed to Dimedrol injection and its harmful health consequences in prisons.

The narrative that conflates MMT with Dimedrol injecting and its harmful effects [22] is especially damaging to public health efforts to implement MMT as an intervention for HIV prevention and harm reduction. Methadone has been a global mainstay of treatment for opioid use disorder for over 40 years. A dose–response curve indicates a minimal dose of 30 mg to prevent symptoms of opioid withdrawal and craving and to effect positive health outcomes, and doses must be individually titrated to reduce the risk of over-sedation and respiratory depression [29]. Numerous prior studies have suggested that when methadone dosing is inadequate to prevent cravings, polysubstance use prevails and contributes to negative health outcomes [30]. One possible explanation for Dimedrol injection reported here is that patients are experiencing suboptimal methadone dosing and that prison medical staff should be doing more to screen for concurrent drug use and monitor drug craving in patients receiving methadone. Individuals who use Dimedrol concurrently with MMT often do so intentionally seeking a high, suggesting MMT in the absence of other supportive services or interventions insufficiently manages symptoms of substance use disorders, including impulsivity. Although people on MMT in Kyrgyz prisons are excluded from heroin distribution, they may use Dimedrol along with MMT to achieve euphoria, combat the boredom inherent to life in prison [31], or self-medicate mood disorders like depression or anxiety [32]. Although these motivations were scarcely mentioned by our key informants, they offer a compelling potential explanation for Dimedrol injection in this context and underscore the need for psychiatric care in these settings.

One way to curb Dimedrol injecting is by attempting to reduce supply, for example by intervening to reduce delivery of Dimedrol from the outside and creating a climate of health and wellness [8]. If we are to apply a purely harm reduction lens to the problem of Dimedrol injecting in Kyrgyz prisons, however, then the solution is not to (attempt to) banish Dimedrol entirely but rather to expand NSP access to support safe injecting practices because many MMT patients are not offered NSP. It is unclear whether crushed Dimedrol tablets, even if mixed with sterile water and injected with sterile equipment, would ever be safe to inject given the seeming toxicity of the substance to veins. Liquid Dimedrol is not widely available in Kyrgyz prisons and might be more difficult for people to conceal, though theoretically potentially safer to inject. PWID in Kyrgyz prisons need low barrier access to urgent medical services for evaluation and management of skin and soft tissue infections, voluntary and confidential HIV testing, and education about the potential physical and psychological harms of Dimedrol injecting. None of these interventions will be successful without the buy-in of formal and informal prison leaders, so a major challenge now is to develop and implement these interventions in a way that is meaningful and sustainable given the particularities of criminal subculture in Kyrgyz prisons. Structural interventions are required given how Dimedrol is situated as part of the informal governing and day-to-day survival of people in prison.

This study has some limitations. Most prisoner participants described the behaviors of other prisoners, rather than their own behaviors, which may reflect their lack of comfort in speaking about behaviors that could be construed as deviant or criminal in a prison setting. Participant statements about Dimedrol injection as “pervasive” or “widespread” in these settings should be interpreted cautiously and considered as much for what they say about the concerns of individuals witnessing and narrating these behaviors. Further studies are needed to quantify the frequency of Dimedrol injection and its sequelae, in Kyrgyz prisons and elsewhere. It is likely that antihistamine injecting is more prevalent than reflected in the existing literature because it is legal (though banned in prisons) and, unlike narcotics, obtained without a prescription. Although some disclosure about illicit behaviors by people in the prison environment may have been limited by fear of surveillance, we conducted interviews with people in prison at every level of the social hierarchy and people outside of prison in the community where there are fewer potential repercussions of disclosure. It is unclear how findings would have differed if the interviews were not audio-recorded. Key informant participants and stakeholders were purposively recruited, which may have unintentionally introduced selection bias and limit generalizability to other prisoners or PWID in the EECA, though that was not the intention of this qualitative study [33]. Finally, most prisoner participants in this study were incarcerated in a male prison. Their views and experiences may differ from those of people incarcerated in a female prison. The vast majority of people in prison (> 95%) are male and injection drug use in Kyrgyzstan is about twice as prevalent in male prisoners (38.3%) than in female prisoners (16.0%) [5]. Women participants in our study rarely described Dimedrol injecting in prison, and thus only one woman’s descriptions of Dimedrol were excerpted here, perhaps because heroin is less widely available in the women’s prison and thus methadone was more socially acceptable.

## Conclusion

Some persons receiving methadone treatment in Kyrgyz prisons also may inject crushed Dimedrol tablets, a non-prescription antihistamine that is banned in prison, to achieve a state of euphoria. Dimedrol injection is asserted to cause devastating physical and mental health effects, including psychosis and necrotic injury. The visible wounds of Dimedrol injection figure prominently in narratives about methadone treatment inside prison. Prisoners who use heroin, which is tightly controlled according to the rules of criminal subculture, do not use Dimedrol, lending heroin a more positive image within prisoner society. People who inject Dimedrol are a foil for the “upright” or “virtuous” prisoner who chooses heroin over methadone. The persistence of Dimedrol injection within these settings is a potentially serious threat to individual health and harm reduction within Kyrgyz prisons and elsewhere in EECA region.

## Data Availability

The datasets used and/or analyzed during the current study are available from the corresponding author on reasonable request.
